# 

**Published:** 1869-02

**Authors:** 


### TO POST-MASTERS AND CLUBS.

GREAT INDUCEMENTS! !

Seven copies of HALL'S JOURNAL OF HEALTH for 1869 will be mailed to one address for Five Dollars, sent at one time, or six copies for Five Dollars, to different addresses.

Write your name plainly, and be sure to give the State, County and name of the Post-Office. It is safest to send money in the form of a Post-Office Order or Bank Check, payable to the order of W. W. HALL, 176 Broadway, New York. Let all subscriptions begin with the January Number.

A QUESTION.—Would you like to subscribe for HALL'S JOURNAL OF HEALTH for 1869, Price $1 50? Or if you wish to present a copy to your clergyman, to a neighbor, relative or friend, seven copies will be sent for Five Dollars, paid at one time, addressed to "Hall's Journal of Health," 176 Broadway, New York.

TO OUR EXCHANGES.—You are hereby authorized as an exchange with "HALL'S JOURNAL OF HEALTH," to offer it and your publication for 1869, at the price of your publication alone, you deducting as much from your regular price as the Journal, in proportion; thus, if yours is three dollars and mine is a dollar and a half, each deducting one-third, gives the price of yours; the offer to remain good for sixty days after you first publish it. W. W. HALL, Editor, Publisher and Proprietor,

176 BROADWAY, NEW YORK.

☞ Post-Masters will please distribute these among clergymen; they are sent to you post-paid.

☞ The enclosed Journals of Health are sent to you, post-paid; please distribute them among brother clergymen or other friends. Seven copies for 1869 will be sent to one address for Five Dollars paid at once.

### MONTHLY PUBLICATIONS.

Atlantic Monthly, devoted to Literature, Science, Art and Politics, published by Ticknor & Fields, 124 Tremont Street, Boston, Mass.; office for New York and Brooklyn subscribers, 63 Bleecker Street, New York, a few doors east of Broadway; —$4 a year, single numbers 35 cents.

American Agriculturist, $1 50 a year, 41 Park Row, New York, published by Orange Judd & Co., devoted to all that pertains to the farm, garden and orchard.

Arthur's Magazine, $2. a year, 8I1 Chestnut Street, Philadelphia.

Blackwood's Magazine, or any one of the Reviews, $4; with any one Review, $7. The 4 Reviews, $12. Blackwood and the 4 Reviews, 15.00. 38 Walker Street, west of Broadway. The Leonard Scott Publishing Company, New York.

Beadle's Monthly; A Magazine of To-Day. $3 a year; 118 William Street, near Fulton. American News Co., Agents, 119 and 121 Nassau Street, New York.

Family Treasure; A Religious and Literary Monthly, W. T. Findley, D. D., Editor and Publisher. $2. Three copies to one address, $5. 65 W. 4th Street, Cincinnati, Ohio.

Gardeners' Monthly and Horticultural Advertiser, edited by Thomas Meehan, No. 23 North South Street, Philadelphia. $2 a year.

Godey's Ladies' Book, Vol. 75, edited by Mrs. Sarah J. Hale and L. A. Godey, $3 a year, corner Sixth and Chestnut Streets, Philadelphia, with numerous and beautiful embellishments each month, Fashion plates, Patterns, &c., &c.

Harper's Weekly, $4 a year, in advance, single copies 10 cents. Harper's Magazine, published monthly at Four Dollars a year; postage 24 cents a year; on the Weekly, 20 cents. 331 Pearl Street, New York.

Horticulturist, and Journal of Rural Art and Rural Taste, published at $2.50 a year, or 25 cents singly, by Woodward, 37 Park Row, New York. Established in 1846.

Hours at Home, $1.25, published by T. S. Arthur & Co., Philadelphia.

Half-Yearly Abstract of The Medical Sciences, being an Analytical and Critical digest of the principal British and Continental Medical works in the preceeding six months, by Henry C. Lea, Philadephia. $2.50 a year, single numbers $1.50

Maryland Farmer, devoted to Agriculture, Horticulture, Rural Economy and Mechanic Arts, published by S. Sands, Mills & Co. at $1.50 a year. 21 South Calbert Street, Baltimore, Md.

Merry's Museum, $1.50 a year, 172 William Street, New York.

Mother's Journal and Family Visitant, edited by Miss Caroline O. Hiscox and published by Sheldon & Company, 500 Broadway, New York.

New England Farmer, published by Eaton & Co., 34 Merchants Row, Boston. $1.50 a year, single copies 15 cents; 52 pages, 8vo, monthly.

Sabbath at Home, an illustrated religious magazine for the family, published by the American Tract Society, 28 Cornhill, Boston, and at 13 Bible House, New York, by the Messrs. Broughton & Wyman. $2 a year, or $2 50.for six months.

Packard's Monthly, New-York. $1 a year.

### MEDICAL EXCHANGES (IN PART.)

BOSTON MEDICAL AND SURGICAL JOURNAL, $4 a year, published by David Clapp & Son, 334 Washington street, Boston, Mass. Now in its 75th volume.

BUFFALO MEDICAL AND SURGICAL JOURNAL, $3 a year, editea by Julius F. Miner, M. D., Surgeon to the Buffalo General Hospital. Address Medical and Surgical Journal, Buffalo, New York.

DENTAL COSMOS, a monthly record of dental science, devoted to the interests of the profession, edited by Drs. McQuellan & Zilgler, $2.50 a year, single Nos. 25c. Philadelphia, Pa., 528 Arch street; 658 Broadway, New York; 16 Tremont Row, Boston; 100 Randolph street, Chicago, Ill.

DENTAL REGISTER, Cincinnati, Ohio, published monthly for $3.00 a year, is always well edited and richly merits the patronage of the profession.

HERALD OF HEALTH, and Journal of Physical Culture, $2 a year, single Nos. 20c., published by Miller, Wood & Co., 13 and 15 Laight street, New York. W. Tweedie, 337 Strand, London.

HOMEOPATHIST AMERICAN, edited by James G. Hunt, M.D., $1.50 a year, in advance, conducted by an able corps of editors, Drs. Bowling, Eve, Jones and Bleckie.

MEDICAL EXAMINER, Chicago, Ill., edited by N. S. Davis, M. D., professor of the principles and practice of medicine and of Clinical Medicine, &c., &c., $3 a year.

MEDICAL AND SURGICAL PIONEER, Kansas City, Mo., being a monthly record of medicine and surgery; $4 a year.

MEDICAL JOURNAL, Chicago, Ill., vol. 24, $2 per annum, 169 South Dearborn street; address R. M. Lackey, M.D., P. O. box 2175, Chicago, Ill. Edited by Drs. Holmes, Lyman & Lackey.

MEDICAL REPORTER, a semi-monthly record of medicine and surgery, edited by Drs. J. S. B. Alleyne and O. F. Potter, $3 a year; St. Louis, Mo.

PHRENOLOGICAL JOURNAL, $2 a year, 389 Broadway, New York, by Fowler and Wells.

